# GERIATRIC CONDITIONS AND HEALTH CARE UTILIZATION AMONG OLDER ADULTS LIVING IN SUBSIDIZED HOUSING

**DOI:** 10.1093/geroni/igz038.952

**Published:** 2019-11-08

**Authors:** Sarah E Kler, Sun Young Jeon, Kanan Patel, Christine Ritchie, Krista Harrison, Kali S Thomas, Rebecca Brown

**Affiliations:** 1 Warren Alpert Medical School of Brown University, Providence, Rhode Island, United States; 2 UCSF, San Francisco, California, United States; 3 Providence VA Medical Center, Providence, Rhode Island, United States; 4 University of Pennsylvania, Philadelphia, Pennsylvania, United States

## Abstract

In the US, 1.7 million low income older adults live in subsidized housing. Previous research suggests that subsidized housing residents have poorer health status than older adults in the general community. However, little is known about the prevalence of geriatric conditions. To understand these factors we conducted a retrospective cohort analysis of 11,558 Medicare enrollees ages 65+ who were enrolled in the National Health and Aging Trends Study in 2011 or 2015, including 507 living in subsidized housing and 11,051 in the general community. We compared subsidized housing residents to general community residents across measures of sociodemographics, functional limitations, and geriatric syndromes. We also compared the prevalence of hospitalization, move to a higher level of care, and death within five years. Results suggest that compared to general community residents, subsidized housing residents were more likely to be women (66% vs. 55%, p<0.01), racial/ethnic minorities (50% vs. 18%, p<0.01), and to lack a high school diploma (50% vs. 20%, p<0.01). They also had poorer health status, including higher rates of self-reported functional impairment (difficulty with 2 or more ADLs; 16% vs. 10%, p<0.01), probable dementia (15% vs. 8%, p<0.01), and frailty using the three-level Fried frailty index (55% vs 26%, p<0.01). Subsidized housing residents also had higher rates of hospitalization (29% vs. 22%, p<0.01), move to a higher level of care (4% vs. 3%, p<0.01), and death (10% vs. 7%, p<0.01) compared to community-residing peers. These findings will help inform targeted interventions to improve aging in place for this vulnerable population.

